# The Translational Medicine Professional: A Bridge Between Bench and Bedside?

**DOI:** 10.3389/fmed.2018.00294

**Published:** 2018-10-17

**Authors:** Faekah Gohar, Aisha Gohar, Georg Hülskamp, Otfried Debus

**Affiliations:** ^1^Department of Paediatrics, Clemenshospital, Münster, Germany; ^2^Julius Center for Health Sciences and Primary Care, University Medical Center Utrecht, Utrecht University, Utrecht, Netherlands

**Keywords:** human health, patients, research centers, none-tertiary centers, academic track in translational medicine, Translational Medical Professionals (TMP), translational medicine (TM)

Translational medicine (TM) can be defined as the interdisciplinary application of biomedical research for the improvement of health of patients and society. The focus of TM has so far been largely on the bench-to-bedside rather than bedside-community transition of research. Several “Valleys of Death” in this process have been described, identifying transitional failures that may halt or impede the pathway, which would otherwise lead to the development of medicines, technologies, and/or evidence based practice guidelines. In order to help bridge these gaps, increasing patient-orientated research at each stage could improve the success of projects and increase societal impact. Increasing the accessibility and involvement of patients in TM outside of traditional research centers, such as universities and teaching hospitals, is one crucial pre-requisite. For example, where clinical research units with active links to local universities have been set-up, research participation can be increased. Such non-traditional research centers (NRTCs) might include primary or secondary care services, or even social care institutions. TM professionals (TMPs) from multi-disciplinary backgrounds, with work experience in university or research centers and with experience of TM, could play a vital role in this organizational change. TMPs in NTRCs are well placed to collaborate with local universities, larger research centers and commercial research and development organizations. Exchanging information could benefit all shareholders involved. TMPs can also stimulate the education and innovative thinking that is required for TM to achieve its full societal impact. We discuss the scope of a potential role for TMPs in NTRCs, as well as the possible barriers and difficulties they might face, along with measures that could widen the accessibility of TM outside of the traditional setting.

The European Society for Translational Medicine defines translational medicine (TM) as being an interdisciplinary branch of biomedicine supported by three pillars: bench, bedside and community. It's goal is to improve the health of society by improving disease management, e.g., with new therapies (1).

TM has predominantly focused on the bench to bedside approach, with most research activities being conducted in traditional research centers such as specialist centers and universities. Several “Valleys of Death” in TM or the bench-bedside pathway, defined as the route between drug or technology development (the “bench”) and its integration into clinical care (the “bedside”), have been described (2–4). The valleys represent gaps that impede the pathway, impacting the development of medicines, technologies and/or evidence based practice guidelines. Until now, less focus has been on the third pillar of TM: the involvement of the wider community, or “bedside to community” phase^1^. Multi-faceted organizational changes and innovation, for example in trial design, are required to bridge these valleys as success rates of products that reach the ”end” clinical trial stage remain poor (2, 5, 6).

Increasing patient-orientated research at all stages could improve the success of research projects and increase societal impact. Research practice often focuses on select groups of patients, for example those with rare or financially or academically “attractive” diseases, and who are primarily treated in hospitals either in or linked to traditional research centers. Such organizational factors result in an inherently biased system in many respects, including in the setting of research agendas and allocation of funding for projects. Such factors could potentially explain the limited output of the TM pathway. Optimizing the accessibility for patients outside of traditional research centers is also a crucial pre-requisite to innovating TM for the benefit of the wider society. To tackle this problem, Clinical Research Units (CRUs) to link local universities and hospitals have been set-up. Funding through the European Clinical Research Infrastructures Network (ECRIN) has further encouraged the connection of research institutions including CRUs, also referred to as CTUs (Clinical Trial Units) or CRCs (Clinical Research Centers) into hubs and networks in 14 countries across Europe (7). Accessibility to research participation in other non-traditional research centers (NRTCs) such as primary or secondary care services, and social care institutions, should also be addressed. An onus on research funders to require evidence of early and consistent patient input beginning in the consultation phase could be an additional driver of change.

A range of professionals from basic scientists, laboratory members, regulatory agencies, educational facilities, members of ethics boards, and journals are involved in TM. Professionals with expertise in TM (Tranlational Medicine Professionals, TMPs) from multi-disciplinary backgrounds could play a central role in innovating TM (Figure 1). TMPs in NTRCs are well placed to collaborate with the traditional research centers and shareholders, and can coordinate the exchange of information as well as stimulate education and innovative thinking. While some clinical academic tracks for the training of TMPs exist, they may be informal and without a focus on TM. One example where TM and the training of future TMPs was a strong focus was the European Translational Training for Autoimmunity & Immune manipulation Network (EUTRAIN) research and training as part of the EU Marie Curie Initial Training Network programme (8, 9). Whilst most TMPs remain based in the organizations where they are trained, i.e., university and research centers, many will spend at least some of their training time in NTRCs. Encouraging such TMPs to continue research in such sites would have a dual effect of avoiding these skills going to waste and maximize the extension of TM into NTRCs. TMPs in NTRCs may even face less constraints on their work, for example with the freedom to conduct projects for societal benefit rather than to achieve prestige in terms of high impact publications and big grants, which may be the case in specialist research centers. In NTRCs, incorporating research into daily clinical practice allows the advantages of TM, such as increased job satisfaction and professional development, to also reach a wider group of professionals. However, TMPs in NTRCs face their own challenges, such as the long held misbelief that research activities should be secondary to the provision of good patient care and limited to research centers. TMPs should engage with colleagues to widen education about TM and its fundamental tenet of incorporating society. NTRCs could themselves drive the process by changing the culture to support and nurture the process of research, for example by recruiting staff with a research interest or experience. The scope of a potential role for TMPs in NTRCs, particularly in (1) widening participation and (2) improving collaboration in TM outside of the traditional research setting will be discussed and are summarised in Table 1.

**Figure 1:**
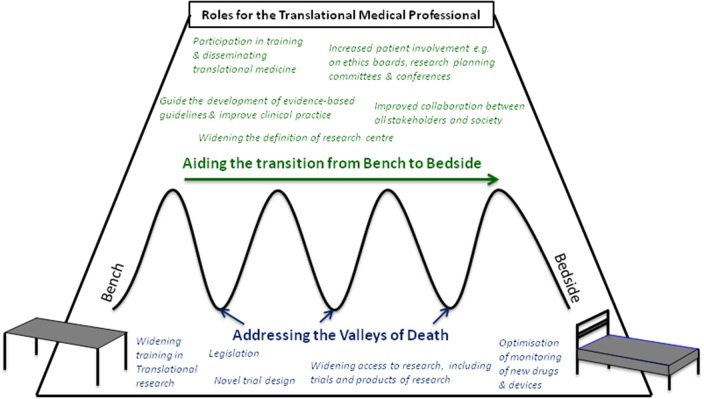
Roles for the Translational Medical Professional in aiding the transition from bench to bedside (green text) and addressing potential points of failure, or “valleys of Death” (blue text).

**Table 1:** Specific roles the Translational Medical Professional could play in shifting the focus of the translational pathway from “bench to bedside” to “bench to society” by (1) widening participation to research and (2) improving collaboration.

**Widening participation**
Encourage involvement in research activities in non-traditional research centers (NTRCs) and other partners including: social care institutions: e.g., hospices, rehabilitation centers, schools, and care homes primary care (general practitioner services) secondary care centers (specialist or teaching hospitals) industry partners universities patient groups ethics research committees Recruit and include patients outside of NTRCs in clinical trials and monitoring of medical devices Encourage the relocation of research and development offices and clinical research units into NTRCs, or take up roles in such centers or work independently but collaboratively with existing centers Take an organizational role in sharing of research facilities such as laboratory facilities Support and encourage the wider inclusion of patient advocates on ethical approval boards and grant approval committees Encourage new grants and apply for existing grants or other benefits, such as awards of a recognition of excellence to research centers that widen participation in TM could be a focus for TMPs in NTRCs. Participation in, and encourage new educational programmes, pre- and postgraduate as well as on-going clinical educational opportunities to address challenges facing translational medicine professionals (TMPs).
**Improving collaboration**
Communication and outreach activities to connect different research partners and participants Organization of collaborative forums and meetings Development and participation in mentorship programmes Setting up and maintenance of shared biobank facilities Organization of the use of specialist research equipment between different centers Mentoring and supporting non-TMP colleagues in realizing the potential personal and wider benefits of TM.

## Widening participation

When research participation is excluded from the majority of NTRCs, a goal of wide societal impact and improvement of health is unlikely to be achieved. All members of society should be seen as potential research participants and receive the opportunity to take part in research (10). All members of society will be affected by healthcare provisions at some point of their life either as recipients of health interventions, or as carers for someone else receiving health care. Therefore, NTRCs should also include social care institutions such as hospices, rehabilitation centers, schools and care homes as well as primary and secondary care centers (10). In addition, some research questions are population based questions, and require broader patient inclusion to be adequately addressed. For this, the support of patient advocacy groups and ethical review boards is also vital, with TMPs supporting the case for widening TM participation in NTRCs.

Longer-term monitoring of drugs and product related adverse effects, for example after clinical trials are concluded or after the acute phase of a disease is over, might be better performed in NTRCs rather than in specialist centers. Whilst the reporting of drug side effects after licensing is encouraged and required in all countries, the monitoring of products is not monitored to the same extent (6). One recent example of the failure of adequate follow up and monitoring of devices is the mounting evidence that mesh used in the surgical management of pelvic organ prolapse has been responsible for many post-surgical complications and that the medical devices (the Mesh) was approved based on weak evidence leading to a large unexpected need for costly post-intervention care (11).

A programme of legislative support and training initiatives is required to support the process of patient engagement (12). Research activities are already being shifted to NTRCs, which can benefit from increased funding streams and patient access and also developing organizational links with local teaching hospitals and commercial research centers (13). Structural changes within NTRC, such as the setting up of research and development offices and facilities for clinical research, are also vital. While their financial set-up may not be under the control of TMPs, TMPs can support their development and help staff them. Clinical research centers often include outpatient facilities with consultation rooms and treatment beds as well as access to a laboratory which can perform basic research procedures such as Real-time PCR and flow cytometry, sample preparation for DNA extraction or serum bio-banking.

To be effective, TMPs should be adequately trained and be inter-disciplinary, including laboratory staff and research coordinators as well as specialist research and clinical nurses and doctors (14, 15). Therefore, a programme of widening participation for TMPs is also required. In the UK, academic clinical fellowships (ACF) during clinical training have improved access to research programmes for trainees. In contrast to the UK, a much greater proportion of medical students in the Netherlands will undertake PhDs during their study or early in their training. In Germany, to obtain the title “Dr. med” a period of research is also usually completed during university study, much akin to intercalated degree programmes in the UK. However, ACFs and most Dr. med. or Ph.D. and research programmes are based in research centers and include little or no focus on TM or inter-disciplinary working. Widening such programmes whether they are pre- or post-graduate based to multi-disciplinary participants and including time in the programme to develop and teach widening participation in research, novel trial design and collaboration and the inclusion of a period of training time in NTRCs is also vital. There is a general consensus that research and TM requires specially trained professionals, and there is increasingly financial and structural support for interdisciplinarity in clinical and research settings. Many universities have developed new institutes with industry partners as well as clinicians and researchers collaborating and now also offer translational study programmes^1^^,^^2^(6). However, one of the largest challenges in widening participation in TM in NTRCs is achieving the organizational changes to support such a transition.

## Improving collaboration

TMPs could foster links between NTRCs and local research centers which excel in a particular field or service by driving collaborations as well as widening research participation. Practical measures may include the organization of regular open meetings, with an open forum to present ideas and updates for new or on-going research projects that could help overcome problems or barriers that projects may be facing. This inter-disciplinary sharing of information could drive innovation and benefit all parties involved, e.g., by pooling potential research participants and sharing access to technology or specialists. Common goals and challenges could help lead to solutions such as the recruitment of a suitable control group. Collaboration between departments from different centers, or even between departments from the same center that may have been unaware of pre-existing research facilities or goals available in-house could be improved upon. Open and equal exchanges of ideas, which is the basis of inter-disciplinary research, opens the door to broader sources of funding. Traditional hierarchies of power, which still often exist in traditional research centers, may also be more effectively challenged when committees are inter-disciplinary. Collaboration between NTRCs and established research centers could also be organized in the form of “outreach programmes” which might include the development of mentorship programmes. Taking an active role in the development and running of such integration and outreach activities could provide career benefits to early-stage TMPs, providing earlier opportunities to undertake leadership roles.

## Challenges facing TMPs

Some challenges facing TMPs focus around accepting the idea of TM in NTRCs. Many TMPs will have trained with a specialist focus. For their new role in NTRCs, TMPs will need to maintain this focus on detail but also develop wider research skills including novel trial design and collaborative work, which takes public health into account. The role of a TMP will comprise many challenges, including that they must work hard in their NTRCs to be seen as effective and successful in both their clinical and research activities. TMPs must also cross barriers such as addressing common misconceptions including that research has no place in clinical training programmes and be able to engage colleagues to also drive good research practices in their workplace (13). The main barrier will be to change perceptions so that research is seen as a part of daily practice in NTRCs and not as a supplementary or a career progress driven activity. TMPs will also need to develop time management skills as well as leadership and delegation if they are to achieve all the activities associated with TM including: teaching, publishing papers, writing research grants. Balancing expectations from colleagues, supervisors and patients will also be vital.

In order to achieve the variety of goals we have discussed as well as to excel in communication and drive innovation, TMPs must be creative—a skill which is difficult to teach and measure. This creativity is fundamental to driving new concepts in the design and practice of trials as well as of medical products and the TM pathway itself (6). TMPs must also use their creativity to develop collaborations with research centers, universities and commercial centers. This can all be achieved with support from colleagues, mentors, and collaborative practices as discussed above.

## Summary

In conclusion, greater focus on the societal aspect in TM is required to tackle the so-called “valleys of death.” The TMP could be a potentially vital driver of innovation and the organizational processes that are required. However, whilst the focus on TM and the number of TMPs might be increasing, TMPs still face multiple challenges but there are many ways in which they can help widen access of TM and improve collaboration within TM.

## Author contributions

FG conceived the study and performed the literature review. All authors contributed to the writing of the manuscript and made substantial contributions to the content and approved the final version.

### Conflict of interest statement

The authors declare that the research was conducted in the absence of any commercial or financial relationships that could be construed as a potential conflict of interest.

## Footnotes

^1^*University of Glasgow - Postgraduate study - Taught Degree Programmes A-Z - Translational Medicine* (Accessed April 21, 2018). Available online at: https://www.gla.ac.uk/postgraduate/taught/translationalmedicine

^2^*Institute of Translational Medicine - University of Liverpool* (Accessed April 21, 2018). Available online at: https://www.liverpool.ac.uk/translational-medicine/about-us/
